# Supplementary material for: Immunomodulation by 4-Hydroxy-TEMPO (TEMPOL) and Dimethyl Fumarate (DMF) After Ventral Root Crush (VRC) in C57BL/6J Mice: A Flow Cytometry Analysis
Source: Biology (Basel). 2025 Apr 25;14(5):473. doi: 10.3390/biology14050473 (PMC12109085; doi:10.3390/biology14050473)
Supplement: Supplementary file 1 [file biology-14-00473-s001.zip › biology-3488186-supplementary.pdf]

Supplementary Material

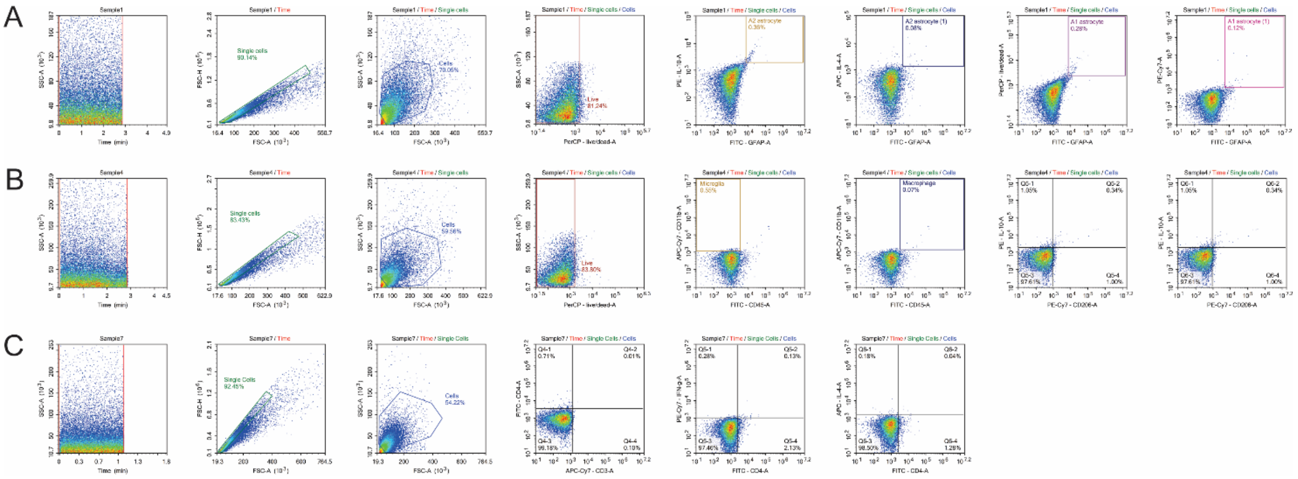

Figure S1. Unstained controls. **(A)** Control for the astrocyte subpopulation. **(B)** Control for the microglia and macrophage subpopulation. **(C)** Control for the lymphocyte subpopulation. Scheme based on the animal treated in the vehicle group at 7 dpi. This method was used for all treatment groups at the three different time points.
